# Influence of Poly-(L-Lactic Acid) Nanofiber Functionalization on Maximum Load, Young's Modulus, and Strain of Nanofiber Scaffolds Before and After Cultivation of Osteoblasts: An *In Vitro* Study

**DOI:** 10.1100/tsw.2009.149

**Published:** 2009-12-16

**Authors:** Jürgen Paletta, Karla Erffmeier, Christina Theisen, Daniel Hussain, Joachim H. Wendorff, Andreas Greiner, Susanne Fuchs-Winkelmann, Markus D. Schofer

**Affiliations:** ^1^ Department of Orthopedics, University Hospital of Marburg, Marburg, Germany; ^2^ Department of Chemistry, University Hospital of Marburg, Marburg, Germany

**Keywords:** electrospinning, nanofibers, tissue engineering, PLLA functionalization, BMP blending, collagen, mechanical stability

## Abstract

The aim of this study was to characterize the influence of functionalization of synthetic poly-(L-lactic acid) (PLLA) nanofibers on mechanical properties such as maximum load, elongation, and Young's modulus. Furthermore, the impact of osteoblast growth on the various nanofiber scaffolds stability was determined. Nanofiber matrices composed of PLLA, PLLA-collagen, or BMP-2–incorporated PLLA were produced from different solvents by electrospinning. Standardized test samples of each nanofiber scaffold were subjected to failure protocol before or after incubation in the presence of osteoblasts over a period of 22 days under osteoinductive conditions. PLLA nanofibers electrospun from hexafluoroisopropanol (HFIP) showed a higher strain and tended to have increased maximum loads and Young's modulus compared to PLLA fibers spun from dichloromethane. In addition, they had a higher resistance during incubation in the presence of cells. Functionalization by incorporation of growth factors increased Young's modulus, independent of the solvent used. However, the incorporation of growth factors using the HFIP system resulted in a loss of strain. Similar results were observed when PLLA was blended with different ratios of collagen. Summarizing the results, this study indicates that different functionalization strategies influence the mechanical stability of PLLA nanofibers. Therefore, an optimization of nanofibers should not only account for the optimization of biological effects on cells, but also has to consider the stability of the scaffold.

### INTRODUCTION

Electrospinning is a simple and effective fabrication technique for producing randomly orientated nanofiber scaffolds. With respect to tissue engineering, these matrices offer great advantages[1,2] due to the fact that they mimic the extracellular matrix[3,4], and allow growth and differentiation of different cell types[5]. These nanofibers can be produced by a broad spectrum of polymers, including biocompatible as well as biodegradable polymers, such as poly(glycolic acid) (PGA), poly(l-lactic acid) (PLLA), poly(ε caprolactone) (PCL), polyurethanes, polyphosphazenes, collagen, gelatin, and chitosan, and copolymers from the corresponding monomers in various compositions[4,6].

PLLA, approved by the FDA, is used in several biomedical applications due to its degradation characteristics and nontoxic degradation products[7,8,9]. Over the last decade, several applications of this polymer in the field of bone surgery have been reported (for review see [7,10,11,12,13,14]).

Therefore, it is feasible to produce and examine nanofibers of this polymer with respect to bone tissue engineering. Osteoblast cell lines like MG63[15,16], osteoblasts[17], as well as human mesenchymal stem cells (hMSC)[15,16], can be cultured and differentiated without visible signs of apoptosis, as demonstrated by fluoresceindiacetate[18]. Nevertheless, a time-dependent analyses of alkaline phosphatase (ALP), collagen I (COLI), and osteocalcin (OC) gene expression indicated an initial down-regulation of genes associated with the osteoblast lineage[19,20]. Using other methods, similar data were presented for PLLA nanofibers[21,22,23] and nanofibers made of other polymers[24,25]. Functionalization of PLLA nanofibers can be a suitable tool in overcoming the proven delay in expression of osteoblast lineage genes. In earlier studies, we demonstrated that the incorporation of growth factors like BMP-2 into the PLLA nanofibers[20] or blending the PLLA with collagen in different ratios[26] is a suitable method in enhancing the osteoinductivity of PLLA nanofiber scaffolds.

When PLLA nanofibers are used in a scaffold, either as a three-dimensional matrix in tissue engineering or as a replacement material *in vivo*, the constructs should be stable to some extent. With respect to bone, cartilage, or tendon repair, they have to resist mechanical deformation, indicating that Young's modulus is an important mechanical property. Furthermore, elasticity and strength of a scaffold are important when used in a musculoskeletal environment for tissue engineering. Therefore, a functionalization of nanofiber scaffolds in order to enhance the biological function only makes sense when mechanical stability of the scaffold is achieved. Only little is known about the influence of such modification on the mechanical properties of the PLLA nanofiber scaffold, so this study was designed in order to elucidate the impact of growth factor incorporation and collagen blending on maximum load, strain, and Young's modulus of the nanofiber scaffolds.

### MATERIALS AND METHODS

#### Construction of Nanofibers and Characterization

The preparation of PLLA nanofibers from dichloromethane (DCM) by electrospinning has been reported in detail earlier[18,19]. Electrospinning of PLLA (Resomer L210, Boeringer Ingelheim Germany) nanofibers from the hexafluoroisopropanol (HFIP) process was performed at a flow rate of 12–14 µl/min, with an applied voltage of 10–18 kV, and a distance of 15 cm from a 4.5% (w/v) polymer solution. The incorporation of BMP-2 has been described in Schofer et al.[20]. Blending of PLLA nanofibers with collagen was performed as reported earlier[26]. All nonwoven nanofibers were collected on a rotating counter electrode (10 cm in diameter) in order to achieve homogenous, randomly orientated, fiber mats. This was important because the alignment had a profound effect on the mechanical properties of scaffolds[27].

Thickness of each nanofiber mat was determined using a torque-limited micrometer screw (Mitutoyo: 0–25 mm, 0.001 mm Nr293-805) applying a surface pressure of 15 $N/cm^2$. Static contact angles of water were measured using the sessile drop method with a G10 Drop Shape Analysis System (Krüss, Hamburg,

Germany) and calculated using Data Physics SCA20 Contact Angle Analyzer Software. For scanning electron microscopy (SEM), samples were sputter coated with gold in an AUTO-306 (BOC Edwards, Crawley, Sussex, U.K.), high-vacuum, coating system and examined in a SEM (S-4100, Hitachi Ltd., Tokyo, Japan) at an accelerating voltage of 5 kV in the SE mode.

### Specimen Preparation and Mechanical Testing

Mechanical testing was performed using a uniaxial testing machine (zwicki-Line, Zwick GmbH KG, Ulm, Germany) with a KAF-TC 200-N load cell. Test specimens of 3.5-mm width, 80-mm total length, and 35-mm gauge length were prepared from the mats using a punch, and then fixed in the machine using rubber-covered metal grips.

Load to failure analysis was done at a crosshead speed of 10 mm/min, without any pretension or preconditioning. Load (N) and extension (mm) were recorded. Young's modulus determination was done by regression between 0.05 and 0.25% strain, and strain was calculated from the displacement of the grips using test expert software (Zwick GmbH KG, Ulm, Germany) at maximum load.

Specimens used in cell culture were washed with phosphate buffered saline (PBS) and then dried in an Exsiccator over copper sulfate for 48 h before mechanical testing.

### MG63 Cell Culture

MG63 (ATCC: CRL-1427) were incubated in Dulbecco's Modified Eagle Medium (DMEM) with low glucose and glutamine (PAA, Linz, Austria), supplemented with 10% fetal calf serum (FCS) from selected lots (Stem Cell Technologies, Vancouver, Canada) and 1% penicillin/streptomycin., at 37°C in a humidified atmosphere (5% $CO_2$/95% air). The medium was changed at 3-day intervals.

For experiments, MG63 were seeded at a density of $3 \times 10^4$ cells/cm² on nanofiber scaffolds in osteogenic differentiation medium according to Jaiswal et al.[28]. The medium was replaced every third day of culture.

### Statistics

All values were expressed as mean ± standard error of at least 10 specimens. Samples breaking in contact with the grips were discarded from analysis. Distribution of the obtained values was analyzed using the Kolmogorov-Smirnov-test. Mean values were compared using Students' t-test or ANOVA, with Bonferroni as a posthoc test. Values of $p < 0.05$ were considered to be significant.

## RESULTS

### Characterization of PLLA and Functionalized PLLA Nanofibers

The average thickness of the nanofiber scaffolds used in this study varied between 0.021 and 0.074 mm, and the density of the scaffolds ranged from 0.206 to 0.414 g/cm³ depending on the solvent as well as on the functionalization technique.

SEM of electrospun nanofibers revealed a three-dimensional, nonwoven network (Fig. 1) with a diameter and contact angle depending on the scaffold as indicated in Table 1. Here, fiber diameter as well as contact angle were comparable to nanofibers used in earlier studies[19,20,26,29].

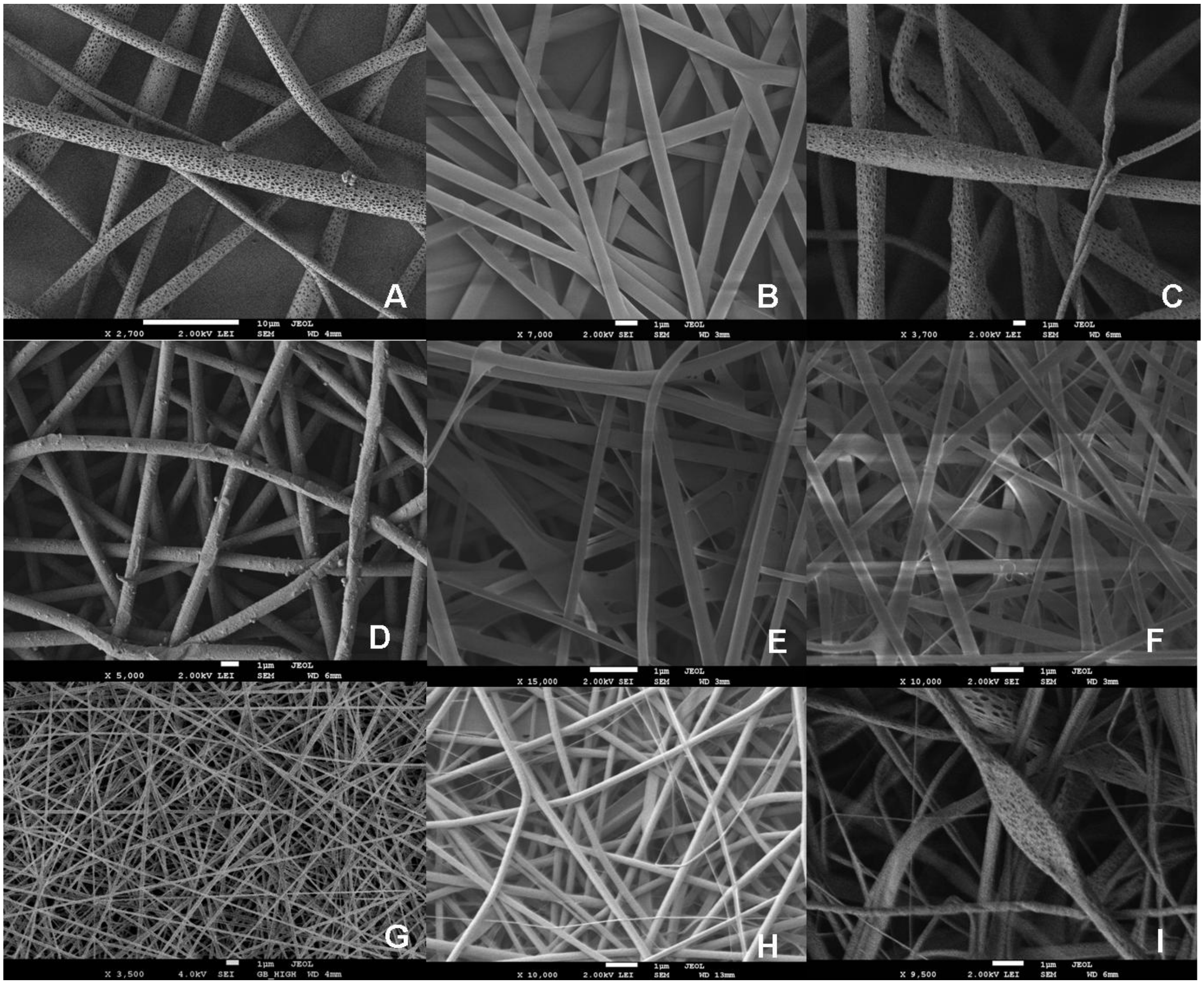

**FIGURE 1.** SEM analysis of PLLA nanofibers after different functionalization procedures. PLLA electrospun from DCM (A) or HFIP (B). PLLA electrospun from DCM (C) or HFIP (D) after incubation in the presence of cells over a period of 22 days. PLLA-COLI blend 1:1 ratio (E) and PLLA-COLI blend 4:1 ratio (F). Same blend supplemented with BMP-2 (G). PLLA electrospun from HFIP supplemented with BMP-2 (H) and PLLA electrospun from DCM supplemented with BMP (I).

### Influence of the Solvent on Stability of the PLLA Nanofiber Scaffolds

In order to elucidate the influence of the solvent used during the electrospinning process, PLLA scaffolds electrospun from DCM or HFIP were stretched to failure. As shown in Fig. 2, the solvents used in this study had no significant effect on maximum load as well as on Young’s modulus. However, strain was up to 10 times higher when nanofiber scaffolds were produced from HFIP as compared to DCM. When cells were cultured on scaffolds electrospun from DCM over a period of 22 days, a significant loss in ultimate failure loads was observed, while strain and Young’s modulus were not affected significantly. In contrast, PLLA nanofibers produced from HFIP showed higher ultimate failure loads, while Young’s modulus was not influenced by the presence of cells in a significant manner. However, a significant loss in strain could be observed after 22 days of cell culture. Nevertheless, in this case, the strain remained higher compared to nanofiber scaffolds electrospun from DCM.

**TABLE 1**
**Characterization of Nanofiber Scaffold used in this Study**

| Polymer | PLLA | PLLA | PLLA after 22 Days of Cell Culture | PLLA after 22 Days of Cell Culture | PLLA-Collagen Blend 1:1 | PLLA-Collagen Blend 4:1 | PLLA-Collagen Blend 4:1 after 22 Days of Cell Culture | PLLA-BMP-Collagen Blend 4:1 | PLLA-BMP-Collagen Blend 4:1 after 22 Days of Cell Culture | PLLA-BSA | PLLA-BMP after 22 Days of Cell Culture | PLLA-BSA |
|---|---|---|---|---|---|---|---|---|---|---|---|---|
| **Solvent** | **DCM** | **HFIP** | **DCM** | **HFIP** | **HFIP** | **HFIP** | **HFIP** | **HFIP** | **HFIP** | **DCM** | **DCM** | **HFIP** |
| Surface | Porous | Smooth | Porous | Smooth | Smooth | Smooth | Smooth | Smooth | Smooth | Porous with spindle-shaped beads | Porous with spindle-shaped beads | Smooth |
| Scaffold thickness [mm] | 0.074 | 0.040 | 0.044 | 0.051 | 0.042 | 0.041 | 0.026 | | 0.023 | 0.021 | 0.026 | 0.043 |
| Scaffold density [g/cm³] | 0.226 | 0.205 | 0.261 | 0.296 | 0.207 | 0.226 | 0.206 | | 0.414 | 0.362 | ND | 0.263 |
| Diameter [µm] | 1.626 | 0.754 | ND | ND | 0.277 | 0.377 | 0.248 | 0.248 | ND | 0.752 | ND | 0.238 |
| Contact angle [°] | 118.1 | 118.4 | <30.0 | 64.9 | 74.2 | 103.2 | 53.7 | | 48.9 | 52.0 | <30.0 | <30.0 |
| Mean weight/ specimen [mg] | 4.7185 | 2.2726 | 3.2331 | 4.2277 | 2.4471 | 2.6117 | 1.5140 | | 2.6873 | 2.1426 | ND | 3.1905 |

ND = not detectable.

### Influence of Protein Incorporation on Stability of the PLLA Nanofiber Scaffolds

Irrespective of the solvent, the incorporation of BMP showed a significant increase in Young's modulus. Furthermore, strain decreased in nanofibers electrospun from HFIP accompanied by an increase in ultimate failure loads. This could not be observed when fibers were electrospun from DCM (Fig. 3).

When cells were cultured on BMP-2–incorporated nanofiber scaffolds over a period of 22 days, the visible integrity of the scaffold stayed intact. However, they were fragile towards manipulation and there was no chance of preparing the specimen for biomechanical testing.

### Influence of Blending on Stability of the PLLA Nanofiber Scaffolds

The blending of PLLA nanofibers with collagen I had no influence on Young's modulus as shown in Fig. 4. Nevertheless, we observed a drastic loss in strain and an increase in ultimate failure loads at least in PLLA-COL 4:1 ratio (Fig. 4).

### Influence of Cell Growth on Stability of the PLLA Blend Nanofiber Scaffolds

In contrast to the PLLA-BMP2 nanofiber scaffold, the PLLA-COLI functionalized scaffolds yielded measurable specimens after incubation with MG36 cells over a period of 22 days. However, the specimens were weakened, as demonstrated, by a decrease in maximum load and strain (Fig. 5.)

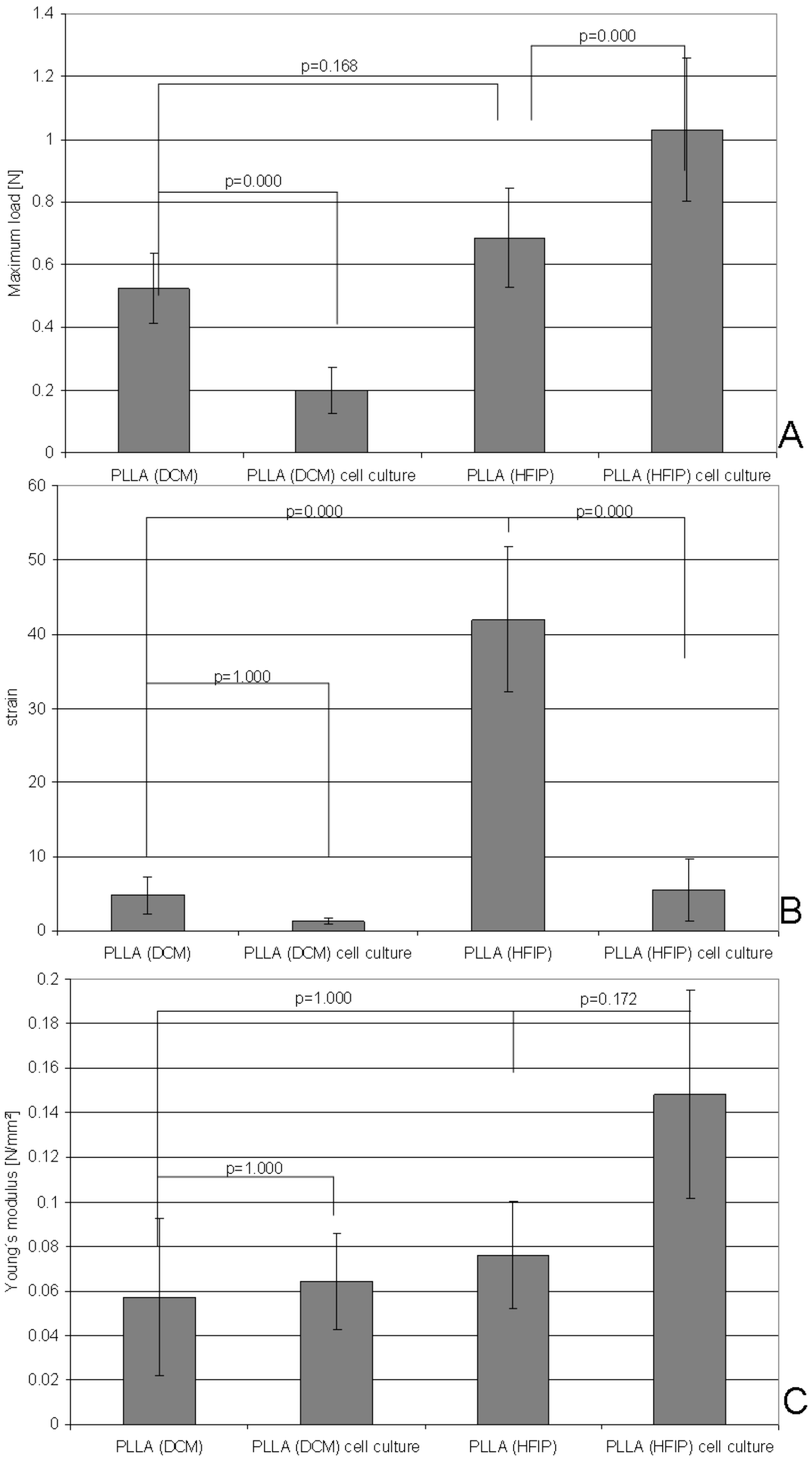

**FIGURE 2.** Influence of the solvent used for the electrospinning of PLLA on maximum load (A), strain (B), and Young's modulus (C) before and after incubation with osteoblast cell line MG63 over a period of 22 days.

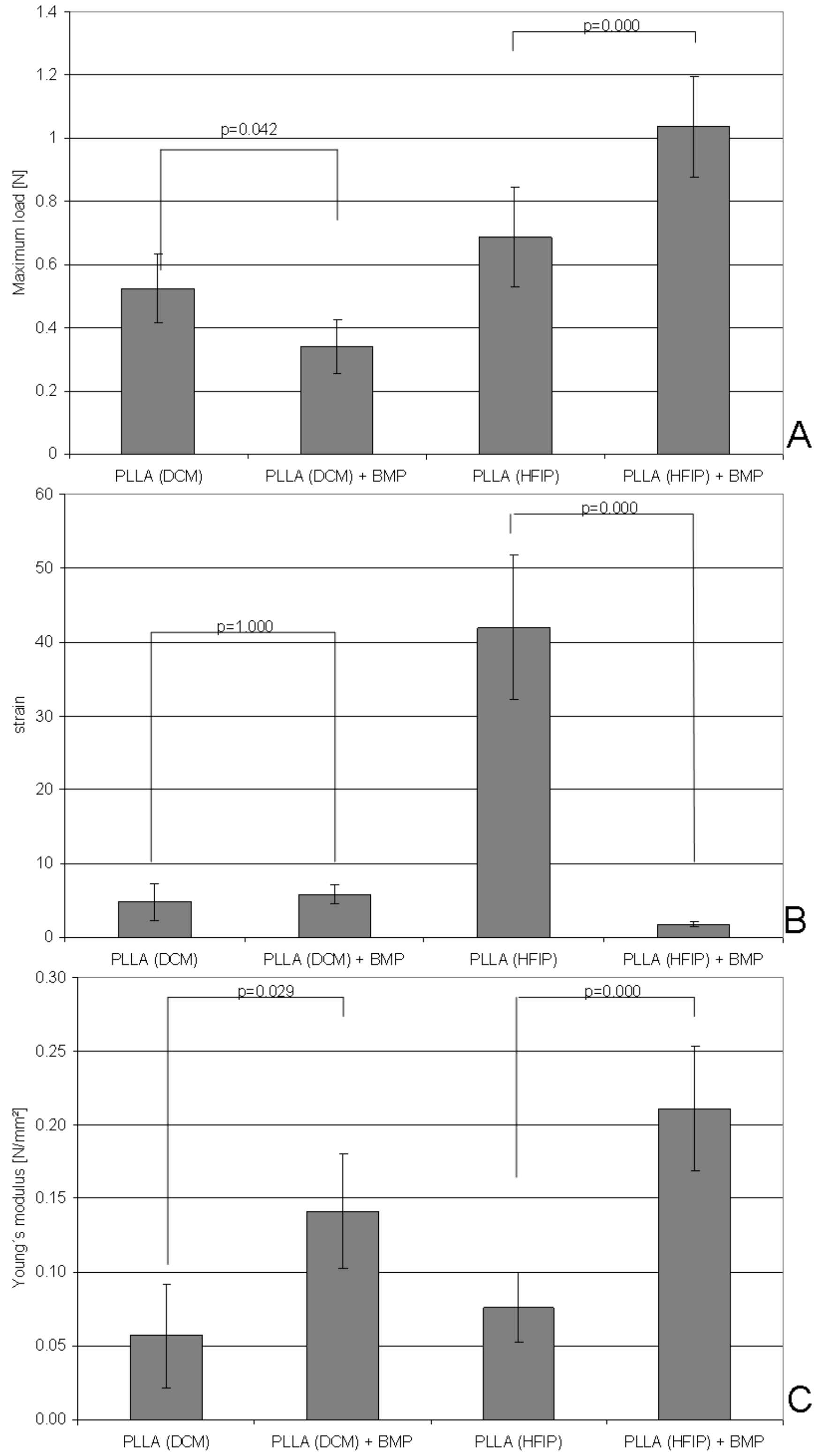

**FIGURE 3.** Influence of the incorporation of BMP into PLLA on maximum load (A), strain (B), and Young's modulus (C).

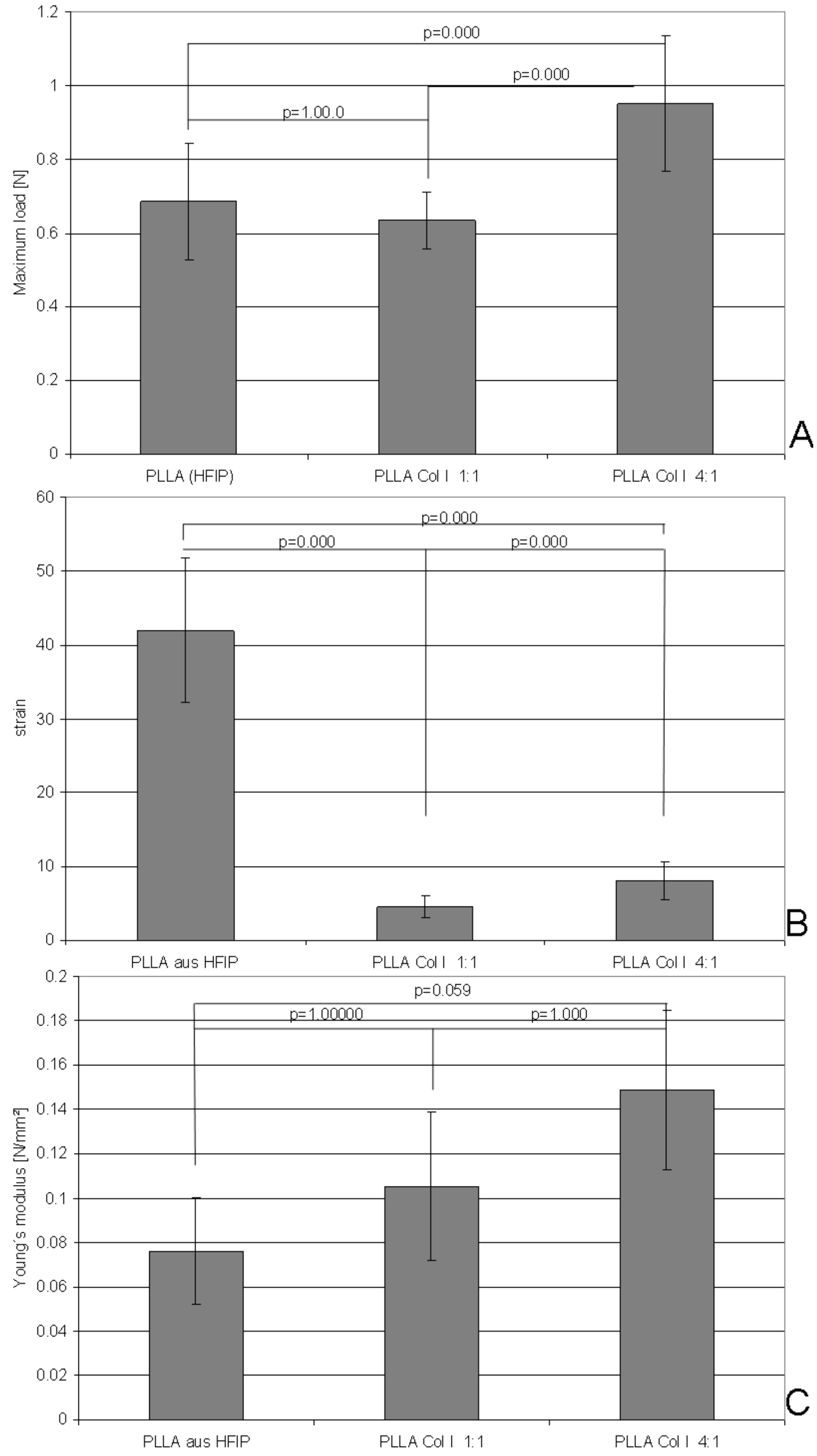

**FIGURE 4.** Influence of blending PLLA with collagen in different ratios on maximum load (A), strain (B), and Young's modulus (C).

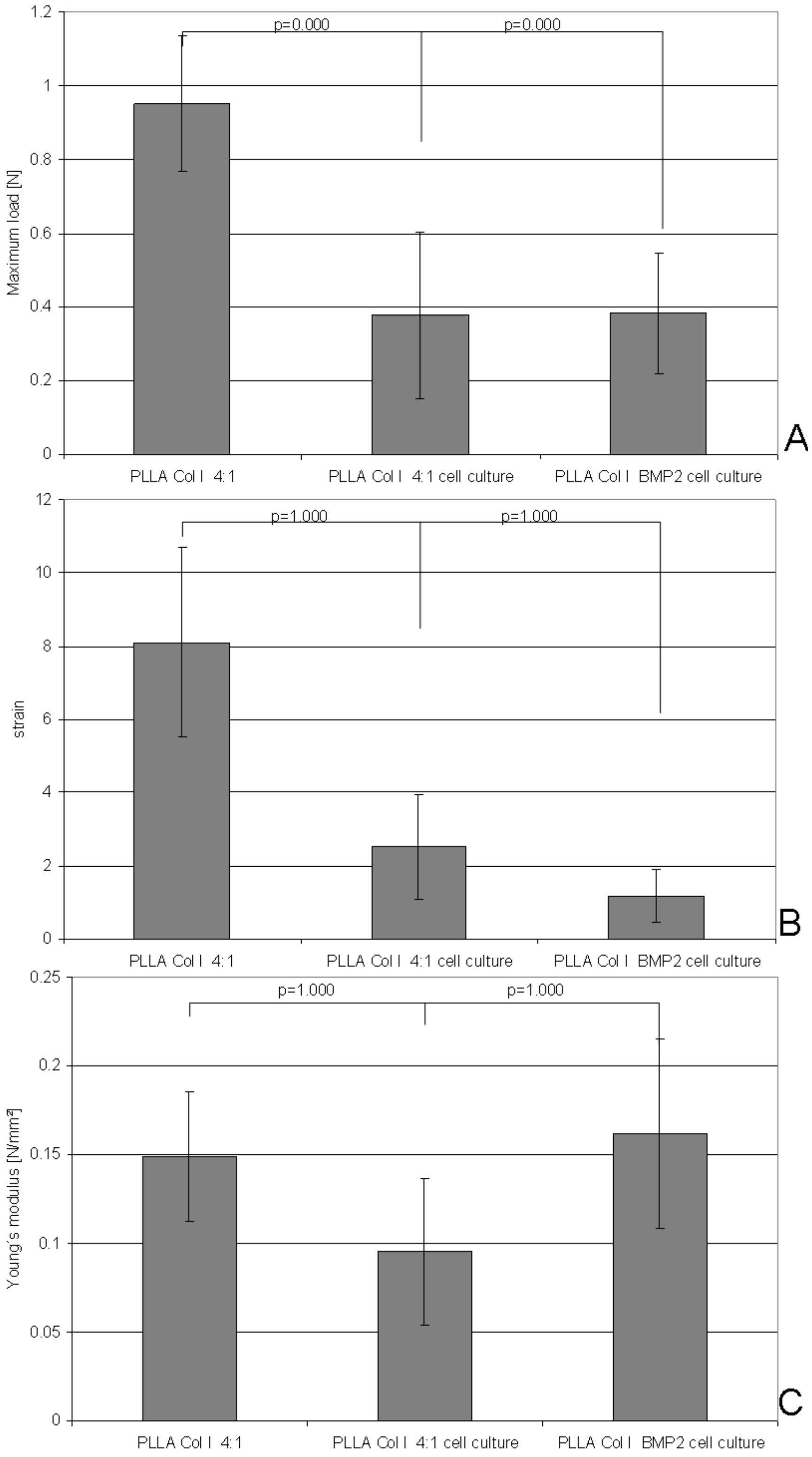

**FIGURE 5.** Influence of cells on PLLA-collagen blend nanofibers on maximum load (A), strain (B), and Young's modulus (C).

### DISCUSSION

PLLA is a biocompatible, biodegradable, and FDA-approved polymer commonly used in pins, screws, or membranes in bone reconstructive surgery[10,30,31,32]. As reported earlier, PLLA can easily be electrospun into a three-dimensional, nonwoven network[33,34]. These constructs are appropriate as a matrix for osteoblast growth as well as for the osteogenic differentiation of hMSC in principle[15,16,18]. The osteoinductive effect of these PLLA nanofiber scaffolds can be enhanced by incorporation of BMP-2[20] or blending with collagen[26]. However, there are no data available concerning the influence of different optimization protocols on the stability of the fiber scaffolds. Within this study, we analyzed the mechanical performance of nonwoven fiber mats. Here, the uniaxial tensile test is most common. As an alternative, atomic force microscopy (AFM) could be used to perform mechanical tests on single nanofibers. However, this will only analyze the impact of functionalization on the fiber itself. With respect to tissue engineering, we believe that the mechanical performance of the scaffold, which comprises more than the stability of the single fiber, is important.

One parameter obviously influencing the nanofiber morphology is the solvent used during electrospinning. Characteristics like boiling point, evaporation speed, and vapor pressure of solvent influence the morphology of the nanofibers. Here, the use of DCM in the electrospinning process resulted in relatively thick fibers with a high porous surface, whereas HFIP leads to thinner and smoother fiber surfaces. The maximum load and especially the strain suggest that the PLLA nanofibers spun from HFIP were more stable, indicating that the solvent plays an important role during electrospinning. The reason for this is unclear, but we assume that differences in the crystalline structure of the resulting nanofibers can be responsible for this effect. Although we did not analyze this, another explanation might be differences in failure mechanisms. It is possible that the porous fiber surfaces got caught during stretching and broke, while the smooth fibers, electrospun from HFIP, were pulled out from the mats, representing a more energy consuming process.

In addition, nanofibers electrospun from HFIP showed a better resistance towards cell growth compared to DCM-spun PLLA nanofibers, although their distensibility was lost in part. Here, the porous structure may be responsible.

Furthermore, the effects of growth factor incorporation on the mechanical stability depend, to some extent, on the solvent. Fibers electrospun from DCM showed spindle-shaped beads in more or less regular intervals, which can explain the increased Young’s modulus. We can speculate that beads, catching together, result in a higher resistance against tension forces. However, this cannot be taken as an explanation for the increased Young’s modulus after the incorporation of proteins in HFIP-spun PLLA nanofibers. Here, electron microscopy analysis revealed no bead formation, but the occurrence of thin ribbons. Therefore, another explanation might be the density and resulting interaction between fibers due to a higher contact area, which seems to differ depending of the solvent. Although not significant, the incorporation of proteins revealed scaffolds of a higher density. Furthermore, there was a correlation of Young’s modulus with the density of the scaffold. We believe that friction forces were higher in scaffolds with higher density, resulting in an increase in the desired parameters.

Besides the incorporation of growth factors into PLLA nanofibers, which has been proven as an effective treatment in order to enhance the osteoinductive properties of the PLLA nanofiber scaffolds[20], the blending of PLLA with collagen has similar effects on the expression of genes associated with the osteoblast lineage[26]. Furthermore, this study demonstrates that the blending of PLLA nanofibers with collagen had little effect on maximum load and Young’s modulus. However, it points in the direction already observed for polyethylenoxide-collagen blends[35].

When subjected to cell culture, these fibers lost part of their stability and strain compared to PLLA fibers electrospun from HFIP, which may be ascribed to a degradation or modulation of the collagen component. However, the blend fibers were more stable after incubation in the presence of cells compared to PLLA or BMP-2 containing fibers spun from DCM.

Summarizing the results, this study indicates that different functionalization strategies influence the mechanical stability of PLLA nanofibers and electrospun fibers in general. Therefore, an optimization of nanofibers should not only account for the optimization of biological effects on cells, but has also to consider the stability of the scaffold.

### ACKNOWLEDGMENTS

This work was supported by the Deutsche Forschungsgemeinschaft (German Research Foundation, Grant No. BO 3065/1-1).
